# Effects of climate factors and Demodex infestation on meibomian gland dysfunction-associated dry eye diseases

**DOI:** 10.1038/s41598-023-50858-y

**Published:** 2024-01-02

**Authors:** Xinran Deng, Wenjie Qi, Shaozhen Zhao, Ruibo Yang, Chen Zhang, Yue Huang

**Affiliations:** 1 Tianjin Key Laboratory of Retinal Functions and Diseases, Tianjin Branch of National Clinical Research Center for Ocular Disease, Eye Institute and School of Optometry, Tianjin Medical University Eye Hospital, Tianjin, 300384 China; 2 Pengzhou People’s Hospital Ophthalmology Department, Chengdu City, 611930 Sichuan Province China

**Keywords:** Climate-change impacts, Conjunctival diseases, Corneal diseases, Eyelid diseases

## Abstract

We examined the effects of climatic factors and Demodex infestations on meibomian gland dysfunction (MGD)-associated dry eye disease (DED) in a cross-sectional study. This study included 123 patients from Tianjin and Chengdu regions, and climate factors and the Air Quality Index (AQI) were recorded for one year. Ocular surface parameters and Demodex infestations were evaluated using various tests. Significant differences in all climatic factors and AQI were observed between Tianjin and Chengdu (*P* < 0.01), and ocular surface parameters also differed significantly between the two regions (*P* < 0.05). Temperature, relative humidity, and precipitation positively correlated with tear break-up time (BUT), meibum gland expressibility, and lid margin irregularity but negatively correlated with lissamine green staining scores (*P* < 0.05). Wind speed and atmospheric pressure positively correlated with corneal fluorescein staining and lissamine green staining but negatively correlated with BUT and lid margin irregularity (*P* < 0.05). AQI positively correlated with DED symptoms and corneal findings but negatively correlated with tear film stability and meibomian gland characteristics (*P* < 0.05). Demodex infestation was only positively correlated with meibum quality scores (*P* < 0.05). Our findings suggest that geographic climates influence ocular surface characteristics in MGD-associated DED, with daily precipitation potentially playing a significant role, and Demodex infestation contributes to meibum gland degeneration.

## Introduction

Dry eye disease (DED) is a multi-factorial ocular inflammatory disorder characterized by tear film instability and hyperosmolarity and neurosensory abnormalities. Its main symptoms include foreign body and burning sensations, blurred vision, and photophobia^1^. In 2007, the Tear Film & Ocular Surface Society (TFOS) Disease Early Warning System (DEWS) divided DED into evaporative and aqueous deficiency subtypes^1,2^. Meibomian gland dysfunction (MGD) is the most widespread cause of over-evaporative DED^3–5^. The prevalence of MGD varies worldwide, with the highest rates observed.in Asia, where the prevalence of MGD-associated DED is estimated at 35.9%^6^. In addition, DED significantly affects the quality of life^7^; it impairs a patient’s ability to read, drive, and work efficiently; causes physical and emotional distress^8^; and places a significant economic burden on society^9^. Moreover, the World Health Organization has emphasized the importance of addressing DED’s risks to human visual health^10^. Therefore, research on DED has significant social value.

Climatic and environmental factors are closely associated with many diseases^11^. As the eyes are the exposed organs, they are influenced by climatic and environmental factors. The DED symptoms, such as dryness, irritation, burning sensation, and redness, tend to exacerbate in dry climatic areas compared to humid climatic areas. Case–control studies conducted in India, Italy, and Brazil have shown a possibility that environmental factors, such as air pollution, wind, humidity, and altitude, may affect the signs and symptoms of DED^12–14^. An extensive population-based survey in the US, involving 606,708 participants, revealed that the risk of DED increased by 13% for each standard deviation increase in atmospheric pressure across different regions. Moreover, high humidity and wind speed were negatively correlated to the prevalence of DED^15^.

In addition to climate change, Demodex infestation has been identified as one of the risk factors in the pathogenesis of MGD-associated DED^4^. Demodex is a parasitic mite that primarily inhabits eyelash follicles and meibomian glands. The pathogenetic mechanisms^16^ of Demodex infestation include direct damage, acting as a vehicle for bacteria, and provoking hypersensitivity; these mechanisms disrupt tear film homeostasis, trigger ocular surface inflammation, and even cause harm to both the ocular surface and meibomian glands.

However, the correlation between climatic variations in different geographical areas, Demodex infestation, and clinical ocular surface parameters remains unclear. Therefore, to further elucidate the pathogenesis and underlying causative factors of ocular surface disorders, more investigations are needed to understand the impact of climatic factors and Demodex infections on ocular surface parameters related to MGD-associated DED. Accordingly, in this study, we recruited individuals with MGD-related DED from two distinct climatic regions to explore the correlations between environmental factors (climate, Air Quality Index [AQI], and weather), Demodex infestation, and ocular surface parameters.

## Results

### Population information

A total of 69 eyes (male: n = 23 and female: n = 46) and 54 eyes (male: n = 10 and female: n = 44) were included in the Tianjin and Chengdu groups, respectively. Table 1 displays the population information for both groups. The chi-squared test was performed to compare the sex distribution between the two groups, and no statistically significant difference was found (*P* = 0.100). Additionally, there was no statistically significant difference in age between the two groups (*P* = 0.416). Moreover, among the MGD-associated DED patients, the mean age of those in Tianjin (44.7 ± 14.1) and Chengdu (46.6 ± 10.6) was similar, and there was no significant difference in the dry eye symptom score (OSDI) between the two groups (*P* = 0.223).

**Table 1:** Demographic characteristics of participants from Tianjin and Chengdu in China.

Parameters	Tianjin area group (n = 69)	Chengdu area group (n = 54)	*P*-value
Sex (Male:Female)^#^	1:2	1:4.4	0.100
Males	23	10	
Females	46	44	
Age (years) ^$^	44.7 ± 14.1	46.6 ± 10.6	0.416

^#^Crosstab analyses were conducted, and results are reported as numbers (percentages). Chi-square tests were used for comparing two regions; ^$^Kruskal–Wallis tests or independent t-tests were used to compare two groups, and results are expressed as mean ± standard deviation. *P < 0.05. **P < 0.01.

### Comparison of climatic factors and AQI between the two different areas

**Figure 1:**
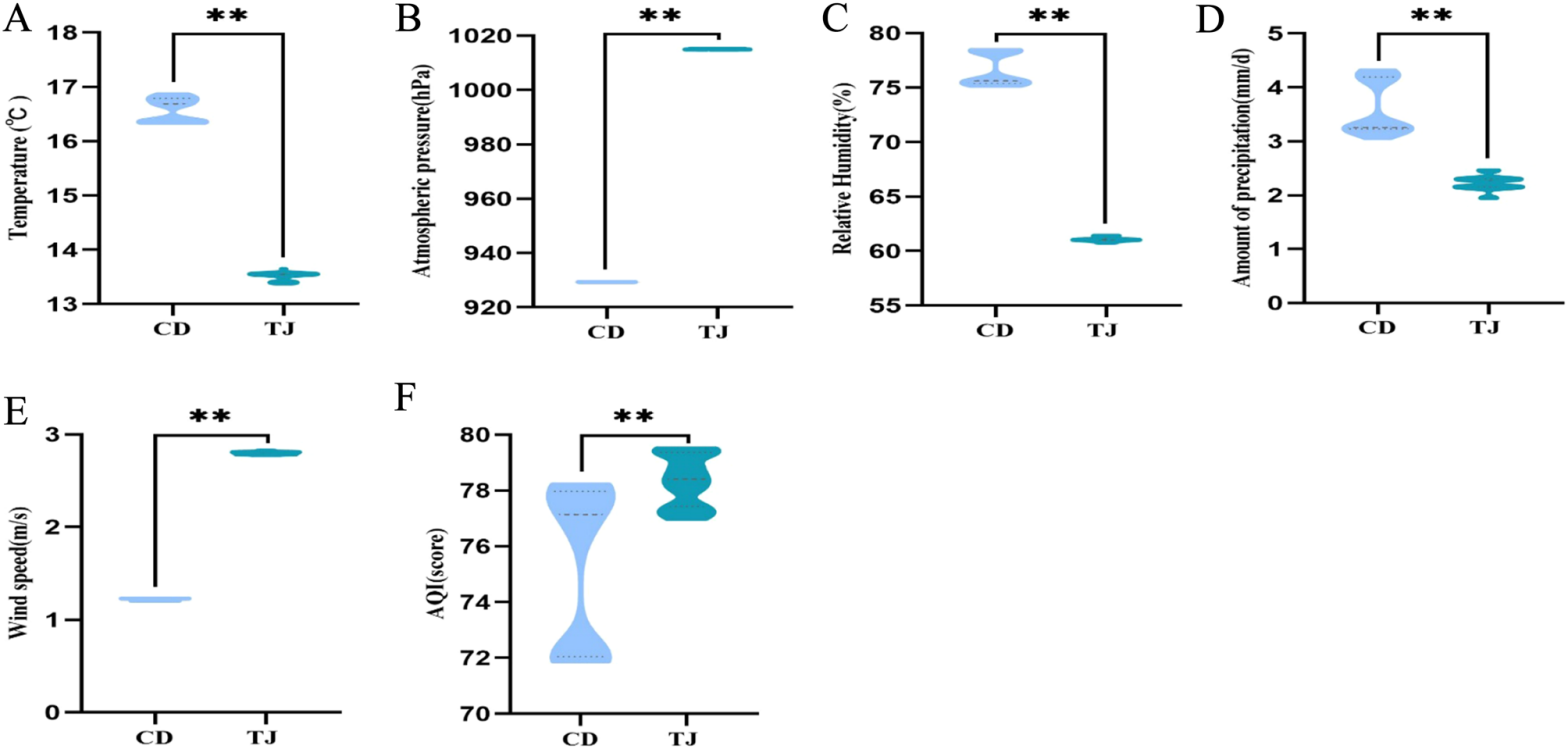
Differences in climatic factors and air quality index between Tianjin (TJ) and Chengdu (CD) areas in China. Temperature (**A**), relative humidity (**C**), and amount of precipitation (**D**) were significantly lower and atmospheric pressure (**B**), wind speed (**E**), and AQI (**F**) were significantly higher in Tianjin than in Chengdu. *P < 0.05. **P < 0.01.

Table 2 and Fig. 1 present a summary of the comparisons of climatic factors between Tianjin and Chengdu areas in China, including temperature (°C), atmospheric pressure (hPa), relative humidity (%), average daily amount of precipitation (mm/d), wind speed (m/s), and AQI. Mann–Whitney nonparametric U test was applied to compare the two groups, and results are recorded as median (25% quantile, 75% quantile). The analysis revealed significant between-group differences in all climatic factors and air pollution indicators (*P* = 0.000), including temperature (°C), atmospheric pressure (hPa), relative humidity (%), amount of precipitation (mm/d), wind speed (m/s), and AQI.

**Table 2:** Various climatic factors and air quality index within one year in Tianjin and Chengdu in China.

Parameters	Tianjin area group (n = 69)	Chengdu area group (n = 54)	*P*-value
Temperature (°C)^&^	13.55 [13.42, 13.56]	16.69 [16.35, 16.79]	0.000**
Atmospheric pressure (hPa)^&^	1015.11 [1015.00, 1015.24]	929.34 [929.30, 929.42]	0.000**
Relative humidity (%)^&^	61.05 [60.96, 61.19]	75.64 [75.38, 78.41]	0.000**
Amount of precipitation (mm/d)^&^	2.27 [2.15, 2.30]	3.26 [3.23, 4.19]	0.000**
Wind speed (m/s)^&^	2.80 [2.79, 2.81]	1.22 [1.21, 1.23]	0.000**
AQI^&^	78.41 [77.43, 79.36]	77.14 [72.05, 77.97]	0.000**

^&^Kruskal–Wallis tests or Mann–Whitney nonparametric U tests were used to compare two groups, and results are shown as medians (25% quantile, 75% quantile).

A *P*-value indicating a significant correlation is shown in bold. *P < 0.05. **P < 0.01.

### Comparison of the ocular surface and meibomian gland parameters between the two different areas

**Figure 2:**
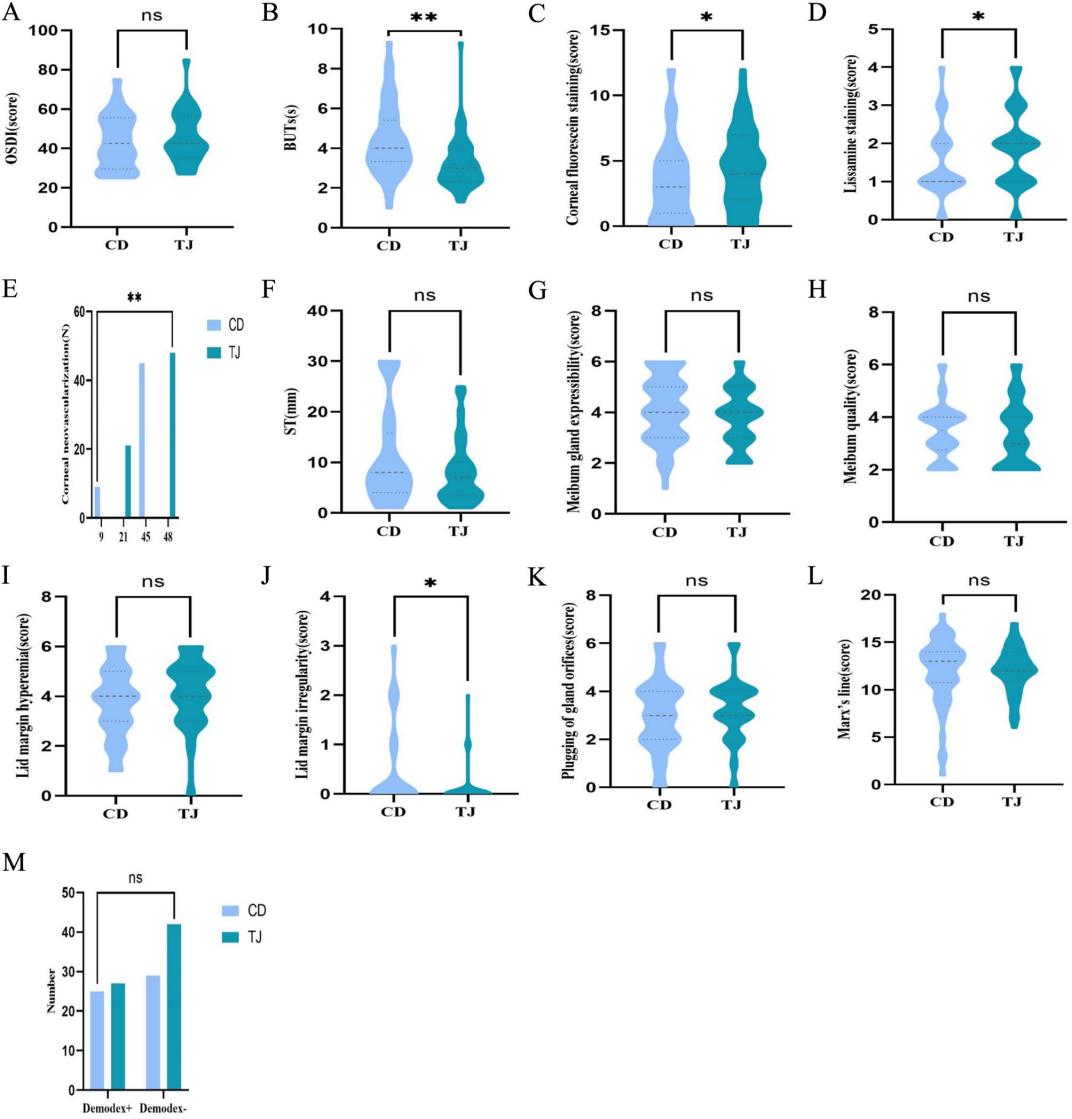
Differences in ocular surface status between Tianjin (TJ) and Chengdu (CD) areas in China. BUTs (**B**) and lid margin irregularity (**J**) were significantly lower and corneal fluorescein staining (**C**), corneal neovascularization (**E**), and lissamine staining (**D**) were significantly higher in Tianjin than in Chengdu. There were no significant differences in ocular surface indicators, such as OSDI (**A**), ST (**F**), meibum gland expressibility (**G**), meibum quality (**H**), lid margin hyperemia (**I**), plugging of gland orifices (**K**), Marx’s line (**L**), or percentage of Demodex infestation (**M**) between the two areas. Ns, *P* > 0.05; *P < 0.05; **P < 0.01.

The comparison of the ocular surface and meibomian gland parameters between Tianjin and Chengdu areas in China is summarized in Table 3 and Fig. 2. Significant differences were observed in several eye examinations (*P* < 0.05), including tear break-up times (BUTs), corneal fluorescein staining (CFS) score, Lissamine staining score, corneal neovascularization, and lid margin irregularity (%); however, no significant differences were found in the Schirmer I test (*P* = 0.126), meibum gland expressibility (*P* = 0.074), meibum quality (*P* = 0.465), lid margin hyperemia (*P* = 0.100), plugging of gland orifices (*P* = 0.204), or Marx’s line (*P* = 0.647) between two areas. Particularly, no significant difference was detected in the percentage of individuals infected by Demodex between the two areas (*P* = 0.465).

**Table 3:** Ocular surface parameters and meibomian gland index in different areas in China.

Parameters	Tianjin area group (n = 69)	Chengdu area group (n = 54)	*P*-value
OSDI (score)^&^	42.50 [36.46, 56.25]	42.50 [29.54, 55.56]	0.223
BUTs (s)^$^	3.22 ± 1.24	4.40 ± 1.77	0.000**
CFS (score)^&^	4.00 [2.00, 7.00]	3.00 [1.00, 5.00]	0.038*
lissamine staining (score)^$^	1.84 ± 1.02	1.46 ± 0.95	0.038*
CNV .n(%)^#^	16.67	30.43	0.000**
ST (mm)^&^	7.00 [3.50, 10.00]	8.00 [4.00, 15.75]	0.126
MGE (score)^&^	4.00 [3.00, 5.00]	4.00 [3.00, 5.00]	0.074
Meibum quality (score)^&^	3.00 [2.00, 4.00]	3.50 [2.75, 4.00]	0.465
LMH (score)^&^	4.00 [3.00, 5.00]	4.00 [3.00, 5.00]	0.100
Lid margin irregularity (score)^&^	8.70	25.93	0.000**
Plugging of gland orifices (score)^&^	3.00 [2.50, 4.00]	3.00 [2.00, 4.00]	0.204
Marx’s line (score)^&^	12.00 [11.00, 14.00]	13.00 [10.75, 14.00]	0.647
Demodex, n(%)^#^	60.87	53.70	0.465

Lissamine staining, Lissamine green staining with Oxford score; CNV, Corneal neovascularization; ST, Schirmer’s I test; Meibum quality, Meibum scores of quality; MGE, Meibum gland expressibility; LMH, Lid margin hyperemia. Continuous variables are expressed as mean ± standard deviation/median (25% quantile, 75% quantile).

^#^Crosstab analyses were performed, and results are reported as numbers (percentage). Chi-square tests were used for comparing two regions; ^$^Kruskal–Wallis tests or independent t-tests were utilized to compare two groups, and results are expressed as mean ± standard deviation;

^&^Mann–Whitney nonparametric U tests were used to compare two groups, and results are presented as median (25% quantile, 75% quantile).

A *P*-value indicating a significant correlation is shown in bold. *P < 0.05. **P < 0.01.

### Correlations among climatic factors, AQI, Demodex infection, ocular symptoms, and signs

Table 4 summarizes the relationships between climatic factors, air pollution indicators, Demodex infection, and various eye parameters during long-term exposure (one year). OSDI scores were positively correlated with the amount of precipitation and the AQI. BUTs and lid margin irregularity showed positive correlations with temperature, relative humidity, and amount of precipitation but negative correlations with atmospheric pressure, wind speed, and AQI. The CFS score was positively related to wind speed and AQI but negatively related to the amount of precipitation. The lissamine staining score was positively associated with wind speed and AQI but negatively associated with temperature, relative humidity, and amount of precipitation. Corneal neovascularization demonstrated a positive correlation with the AQI but a negative correlation with the amount of precipitation. Schirmer I test results were negatively correlated with atmospheric pressure. The meibum quality score exhibited a positive correlation only with Demodex infection. Meibum gland expressibility showed a positive correlation with relative humidity and amount of precipitation but a negative correlation with AQI. Plugging of gland orifices was positively related to atmospheric pressure but negatively related to temperature and AQI. Marx’s line showed a positive correlation only with the AQI.

**Table 4:** Correlations between ocular parameters, climatic factors, and AQI data during long-term exposure.

Ocular parameters	Temperature (°C)	Relative humidity (%)	Atmospheric pressure (hPa)	Amount of precipitation (mm/d)	Wind speed (m/s)	AQI	Demodex
	r	r	r	r	r	r	r
OSDI	0.002	− 0.123	0.085	− 0.205*	0.157	0.226*	0.117
BUTs	0.297**	0.296**	− 0.307**	0.378**	− 0.321**	− 0.286**	− 0.053
CFS	− 0.116	− 0.166	0.168	− 0.217*	0.179*	0.195*	0.098
Lissamine staining	− 0.181*	− 0.178*	0.099	− 0.177*	0.187*	0.228*	− 0.011
CNV	− 0.096	− 0.171*	0.163	− 0.261**	0.113	0.195*	− 0.021
ST	0.042	0.090	− 0.178*	0.150	− 0.148	− 0.140	− 0.018
Meibum quality	0.048	0.093	− 0.047	0.111	− 0.049	− 0.145	0.194*
MGE	0.051	0.181*	− 0.107	0.261**	− 0.097	− 0.229*	0.077
LMH	− 0.155	− 0.127	0.170	− 0.163	0.045	0.066	0.034
Lid margin irregularity	0.218*	0.280**	− 0.322**	0.288**	− 0.240**	− 0.191*	− 0.034
Marx’s line	0.177	− 0.025	0.035	− 0.070	− 0.006	0.249**	0.039

The F (95% confidence interval) indicating a significant correlation is shown in bold. *P < 0.05. **P < 0.01. Two-tail for Spearman correlation analysis.

## Discussion

Climate factors have significant impacts on human health and are intimately involved in the development of various diseases^11,17^. While the influence of climatic factors on human health has predominantly been investigated in relation to the human respiratory and immune systems, the influence of climatic factors on ocular surface diseases, such as MGD-associated DED, blepharitis, and blepharitis-related keratoconjunctivitis, has rarely been explored^18,19^. Notably, a study by Sanghyun Nam et al. retrospectively examined the correlation between air pollution and the prevalence of conjunctivitis in South Korea^18^. In our present study, we recruited patients with MGD-associated DED from two regions (Tianjin and Chengdu) with significant differences in climatic factors, and the relationship between the ocular surface manifestations and climatic factors, AQI, and Demodex infestation was analyzed. The tear film stability, indicators of ocular surface epithelial barrier damage, and relevant indicators of lid gland and ocular surface characteristics were observed and analyzed between the two regions.

Chengdu is situated in the southwestern part of China, specifically in the western region of the Sichuan Basin, and it has a humid, subtropical monsoon climate. In contrast, Tianjin, situated in the northern part of China, lies to the east of the North China Plain, and it has a warm temperate, semi-humid continental monsoon climate. Both cities have large populations with similar demographics. However, there were significant differences in climatic factors and AQI, which reflect China's primary climatic characteristics. Temperature, relative humidity, and precipitation levels were significantly lower in Tianjin than in Chengdu, indicating that Tianjin tends to be colder and drier than Chengdu. In contrast, atmospheric pressure, wind speed, and AQI were significantly higher in Tianjin than in Chengdu, suggesting that Tianjin has higher wind speed, higher atmospheric pressure, and more severe air pollution than Chengdu.

Our study found significant differences in the ocular surface features of MGD-associated DED patients between the two regions. Patients in Tianjin had significantly shorter BUTs than those in Chengdu, indicating that patients in areas with lower temperatures and humidity had poorer tear film stability. Moreover, parameters assessing ocular surface damage, such as CFS and Lissamine staining, were significantly higher in patients from Tianjin than in those from Chengdu, signifying more severe ocular surface damage in cold and dry climates than in warm and humid climates. Additionally, lid margin irregularity scores also demonstrated statistically significant differences between the two regions, with patients from Chengdu scoring higher than those from Tianjin. The finding suggests that patients with MGD in warm and humid areas are more likely to exhibit shallow sulcus-like changes in lid margin morphology. Conversely, no significant differences were found in ocular surface indicators representing the inflammatory status of the lid gland between the two regions. Furthermore, there was no statistically significant difference in the rate of Demodex infection between patients from these two regions.

To clarify the influence of climatic factors on ocular surface parameters in patients with MGD, we conducted a correlation analysis between climatic factors and ocular surface parameters at both study locations. Our findings revealed a significant association between the stability of the ocular surface tear film and climatic factors. Moreover, BUTs were positively related to temperature, humidity, and precipitation, suggesting that ocular surface tear film was more stable in individuals residing in areas with high temperature, relative humidity, and precipitation for an extended period. These findings are consistent with those reported in the study conducted by Hao et al.^20^, which demonstrated that lower temperature led to shortened BUTs, increased neovascularization of the eyelid margin, enhanced plugging of the gland orifices, and worsened meibum gland expressibility. We hypothesize that prolonged exposure to higher ambient temperatures may lead to a more relaxed and spread lid gland orifice, reduced coagulation of the lid gland fluid, lower viscosity after drainage from the lid gland orifice, and a more stable lipid layer in the tear film. Consistently, Borchman et al.^21^ also found that increased temperature led to changes in the conformation of the hydrocarbon chains, facilitating the drainage of lid lipids from the lid gland and enhancing the hydration of the tear fluid with polar lipids, thereby increasing the surface tension of the tear film. In addition, increased relative humidity and precipitation reduce water evaporation from the ocular surface, thus improving the stability of the tear film. In contrast, we found that BUTs were negatively correlated with atmospheric pressure, wind speed, and AQI; the findings suggest that high atmospheric pressure and wind speed may accelerate the volatilization of water in the tear film, and air pollution particulate matter could stimulate inflammatory responses on the ocular surface, potentially altering the concentration of inflammatory factors in the tear film. Further, our study suggests that in addition to known risk factors, such as wind speed, humidity, and temperature, average daily precipitation is a more sensitive protective factor for DED. Taken together, these findings suggest that managing environmental exposure could be crucial in mitigating symptoms and enhancing the quality of life for individuals with DED.

Ocular surface epithelial barrier function was found to be significantly correlated with climatic factors. CFS, Lissamine staining, and corneal neovascularization are important indicators of the structural integrity of the epithelial barrier^22^. We found that the CFS was significantly correlated with wind speed, AQI, and the amount of precipitation. High wind speeds and exposure to heavy air pollution can lead to corneal epithelial cell damage, whereas increased precipitation has protective effects on the corneal epithelium. The positive correlation between Lissamine staining and wind speed or AQI further supports our conclusions regarding the association between CFS and climatic factors. Moreover, lissamine staining was negatively correlated with the amount of precipitation, temperature, and relative humidity, suggesting that higher precipitation levels, temperature, and relative humidity might mitigate corneal and conjunctival epithelial cell loss to some extent. Corneal neovascularization, which aggravates ocular surface damage, exhibited a significant negative correlation with the amount of precipitation and a positive correlation with AQI. Residents in regions with higher precipitation levels experienced less ocular surface epithelial cell damage, while those in areas with higher air pollution exhibited more ocular surface epithelial cell damage and a higher likelihood of developing corneal neovascularization.

The lid margin irregularity score serves as an indicator of the altered morphology of the lid margin. Our study revealed a significantly positive relationship between lid margin irregularity and temperature; higher temperatures were associated with more pronounced changes in lid margin morphology, whereas atmospheric pressure, wind speed, and AQI exhibited a negative correlation with lid margin irregularity. These findings are consistent with the solute gradient hypothesis proposed by Bron et al.^23^. According to this hypothesis, as temperature increases, the evaporative loss of water from the tear meniscus of the eyelid increases, creating a solute gradient in the profile and leading to an increase in the molar concentration of tears at the apex of the peripheral tear meniscus, which exceeds the solute volume at the mucocutaneous junction (MCJ). Previous studies have shown that high osmotic pressure in the tear meniscus induces keratinization of human corneal epithelial cells^24^. Additionally, increased molar concentrations in the tear meniscus intensify the inflammatory response in the MCJ. Consequently, the increased molar concentration of the tear meniscus and the enhanced concentration of inflammatory proteins at the tip of the tear meniscus contribute to the formation of a ‘trough’ of cell death near the MCJ, which over time depletes repair mechanisms at the eyelid margin, resulting in shallow sulcular changes at the lid margin.

The Marx’s line score serves as an important indicator of the inflammatory response at the lid margin. Our study revealed positive associations between Marx’s line scores and AQI levels only, suggesting that air pollution particles could promote inflammatory responses in the lid gland orifice. This phenomenon can be explained by the tear meniscus solute gradient hypothesis^23^. Marx’s line represents a physiological line at the lid margin that can be stained by fluorescein and lissamine green^25^, reflecting the absence of polysaccharide-protein complexes, such as MUC-16 and galectin-3, which can block the entry of dyes into the surface epithelium. Air pollutant particles trigger inflammatory responses on the ocular surface and lid margin, resulting in elevated concentrations of inflammatory proteins in the tear fluid; this process is further amplified by the evaporation of tears from the ocular surface, leading to higher concentrations of pro-inflammatory proteins (e.g., interleukin-1 beta, interferon-gamma, tumor necrosis factor-alpha [TNF-α], and matrix metalloproteinase) in the upper section of the tear meniscus than in the middle section of the tear meniscus^23^. Additionally, TNF-α and neutrophil elastase can cause the shedding of polysaccharide-protein complexes, such as MUC-16, from the surface of human lid margin epithelial cells^26^. Over time, these pathological changes contribute to the pathological widening, irregularity, and anterior displacement of Marx’s line.

When examining the effects of climatic factors on the meibum gland, we found that neither climatic factors nor the AQI had a significant effect on meibum gland expressibility or meibum quality. However, we observed that Demodex folliculorum infection was the only in vitro indicator positively correlated with meibum quality; more severe Demodex folliculorum infection was associated with poorer meibum quality and increased lid lipid degeneration. Consistently, Gao et al.^27^ found significant changes in the levels of (O-acyl)–hydroxy fatty acids in the tear films of patients with MGD who tested positive for Demodex. Moreover, earlier studies^28,29^ also reported similar findings: Ocular Demodex infection is significantly associated with meibum scores, lid margin abnormality scores, and meibomian gland dropout, with higher scores observed in Demodex-positive patients. These observations suggest that several mechanisms may contribute to altered meibum quality as a pathophysiological mechanism for exacerbated signs and symptoms in patients with MGD-associated DED. First, Demodex folliculorum primarily inhabits eyelid follicles, leading to direct mechanical damage, epithelial hyperplasia, and reactive hyperkeratosis^30^. Second, it triggers an inflammatory cascade and immune response on the host ocular surface through toxins present both on its surface and within its body, as well as through the bacteria it carries (such as Streptococci, Staphylococci, and Bacillus oleronius)^31^. Interestingly, we found no variability in the prevalence of Demodex infection between patients in the two locations.

It is widely recognized that Demodex infestation may be associated with factors such as severe air pollution, hygienic conditions, and age^32^. However, our investigation did not find correlations between climatic factors, air pollution, and Demodex infestation. This unexpected finding may be due to the following factors. First, Demodex is a human ectoparasite that resides in the skin follicles, with its survival and proliferation closely tied to the skin and hair health of the host. Consequently, climatic factors and air pollution could exert an indirect influence, rather than a direct one, on Demodex growth and reproduction. In addition, for this study, we chose the participants mainly from the resident population of large metropolitan areas, each with a population exceeding 10 million. The demographics of both cities predominantly feature a multi-ethnic composition with a Han Chinese majority. Moreover, there was no significant age difference among participants from the two regions. According to the Seventh National Census Bulletin of China, both cities are major provincial-level urban centers with comparable healthcare systems. The participants included in the observational group were Han Chinese, middle-income or higher urban dwellers, and they had similar socioeconomic status, healthcare access, and levels of awareness regarding eye health and eyelid hygiene.

Despite these worthwhile findings, there were some shortcomings in this study. First, the sample size was small, and the number of areas observed was insufficient. Although the study included two major cities representing different climates in China, the country’s vast size encompasses diverse climatic regions that were not examined in this study, such as northwest highland climates and southern subtropical climates. Second, the study focused on examining the association between the AQI and eye surface parameters to determine the influence of air pollution on ocular surface parameters in patients with MGD-associated DED. However, it did not perform a detailed analysis of specific air pollution particles and pollutants, such as PM2.5, PM10, SO2, and O3, and other air pollution indicators. Further studies should consider expanding the sample size and including patients from a wider geographical area to further analyze the impacts of atmospheric pollution and climate factors on eye surface disorders. Additionally, conducting a longitudinal study over a more extended period would be beneficial to elucidate the correlation of Demodex infestation with climatic and air pollution factors. Adopting more robust methods, such as extending the observation period and expanding the sample size in longitudinal studies, could yield findings that more accurately reflect real-world scenarios.

## Conclusion

This study revealed that ocular surface characteristics of patients with MGD-associated DED were influenced by geographic climate, and tear film stability was significantly correlated with climatic factors, with daily precipitation levels potentially playing a more significant role. In addition, Demodex infestation was only positively correlated with meibum quality scores. The findings emphasize the importance of considering climatic factors in the management of DED to ensure effective care for affected individuals.

## Methods

### Participants

This study was a cross-sectional study. A total of 123 eyes of 123 patients diagnosed with moderate to severe MGD-associated DED were recruited from the Dry Eye Center of Tianjin Medical University Eye Hospital, Tianjin, and the Ophthalmology Department of Pengzhou People’s Hospital, Sichuan Province, from May to October 2022. In total, 54 eyes with MGD-associated DED from Chengdu and 69 from Tianjin were enrolled in this study. All participants were outpatients aged 18–65 years who had lived in the locality for at least one year and had not traveled outside the observation areas during that time.

Inclusion criteria were as follows: diagnosis of DED according to TFOS International DEWS II and International Workshop on MGD^1,5^. Each participant completed an OSDI questionnaire, and those meeting the inclusion criteria had a score of ≥ 23.0^33^. Exclusion criteria were as follows: smokers; participants suffering from any eye or systemic disease; patients who underwent eye surgery or had ocular trauma within the past 3 months; participants who had used systemic or topical eye medications within the previous 1 month; contact lens wearers; and pregnant or breastfeeding women. This study was approved by both the Institutional Review Board/Ethics Committee of Tianjin Medical University Eye Hospital in China and the Ethical Committee of Pengzhou People’s Hospital, Sichuan Province, China (2022KY-11). The study was performed in compliance with the Declaration of Helsinki, and the authors received informed consent from all participants.

### Experimental procedure

All patients were examined according to a standardized procedure: demographic information collection (including date of birth, sex, sleep time, and electronic use time), OSDI questionnaire, Schirmer’s I test, slit-lamp biomicroscopic examination, BUT, CFS, conjunctival lissamine green staining, meibomian gland assessment, and Demodex examination using a general light microscope. Notably, all the meibomian gland assessments were performed by the same ophthalmologist.

### Collection of data on climatic factors and AQI

Data on climatic factors, including temperature (°C), atmospheric pressure (hPa), relative humidity (%), average daily amount of precipitation (mm/d), wind speed (m/s), and the AQI were obtained from the China Environmental Monitoring Station for two different regions, Tianjin and Chengdu. The AQI consolidates concentrations of all pollutants into a standard single daily figure, which reflects the overall air quality conditions^34^. To investigate the climatic effects on the ocular surface while minimizing the effect of rapid changes in climatic factors over short periods, one year of data on climatic factors and AQI were collected. The average of the four timed measured values taken at 02:00, 08:00, 14:00, and 20:00 was collected as the daily average, and the daily average of the 365 days before enrollment (including the day of enrollment) was used as the long-term climatic data for each patient.

### OSDI questionnaire

The OSDI questionnaire^35^ was utilized to evaluate the severity of symptoms on a scale ranging from 0 to 100. The questionnaire consists of 12 questions. Participants provided responses ranging from 0 to 4 (0: none of the time; 1: some of the time; 2: half the time; 3: most of the time; 4: all the time). Scores ≥ 23 points on the index indicate moderate-to-severe dry eye.

### Schirmer’s I test

Without the administration of anesthetic drops, a Schirmer’s test strip (Tianjin Jingming New Technological Development Co., Ltd, China) was positioned on the central and outer third of the lower lid. Participants were instructed to close their eyes for 5 min, and the level of moisture absorbed by the strip was measured and documented during this period^36^.

### Slit-lamp biomicroscope examination

A comprehensive ocular surface examination was conducted on all patients using a slit-lamp biomicroscope to assess the MGD.

1. *BUTs* A fluorescein test trip (Tianjin Jingming New Technology Development Co., Ltd, China) was applied to the patient’s lower lid conjunctival sac. Participants were instructed to blink several times and then stare straight ahead. The time elapsed from the last complete blink to the appearance of the first randomly distributed corneal black spot (in seconds) was measured three consecutive times, and average values were documented^36^.
2. *CFS* Corneal fluorescein staining was conducted following the assessment of BUTs using the National Eye Institute/Industry grading scale, which ranges from 0 to 15. Each zone of the cornea was assigned a grade as follows: 0, no staining; 1, mild, < 10 scattered staining spots; 2, moderate, between 10 and 30 spots; or 3, severe, > 30 spots, fusion staining, or presence of corneal filaments. A total of five areas were evaluated and recorded^37^.
3. *Lissamine green staining* Lissamine green conjunctival staining was assessed using the Oxford grading system. Following the application of one drop of 1% sodium lysine green strips (Green Glo lissamine green ophthalmic strips, Hub Pharmaceuticals) to the lower conjunctival sac of both eyes, the staining was examined using a slit lamp immediately^38^. The observed staining on the interpalpebral region of the conjunctiva and cornea was compared with the Oxford panel chart to determine the corresponding grade. The same score was assigned based on the Oxford Scale.
4. *Meibum gland expressibility* The five lid glands in the central region of the upper and lower eyelids were individually squeezed at a single stable pressure by a meibomian gland evaluator to observe glandular secretion. The observed secretion was then scored on a scale of 0–3, with the following criteria: 0, all glands expressible; 1, 3–4 glands expressible; 2, 1–2 glands expressible; and 3, no glands expressible^39^. The scores obtained for the upper and lower eyelids were summed to obtain the total score.
5. Meibum quality was assessed in the upper and lower lid glands (the number of glands was not limited) using a 4-point scale as follows: 0, clear fluid; 1, cloudy fluid; 2, cloudy particulate fluid; 3, like toothpaste^40,41^. The scores obtained for the upper and lower eyelids were summed to obtain the total score.
6. *Lid-margin hyperemia* The distribution of abnormal vascularity at the lid margin was examined and photographed using a slit-lamp microscope. The assessment was conducted using the following grading system: 0, no telangiectasia crossing meibomian orifices (Mos); 1, redness observed in the palpebral margin conjunctiva, without telangiectasia crossing Mos; 2, telangiectasia crossing Mos, distributed over less than half the full length of the eyelid; 3, telangiectasia crossing Mos for one-half or more of the eyelid’s full length^42^. The scores of the upper and lower eyelids were summed to give a total score.
7. *Corneal neovascularization* The cornea was classified into four quadrant regions, and scores were assigned based on the following criteria: the number of affected quadrants, the depth of involvement, and the location of the blood vessels. A score of one was assigned to each quadrant. The depth of involvement was categorized as follows: 1, superficial vessel and 2, deep vessel. The location of blood vessels was classified as follows: 1, peripheral vessel; 2, mid-peripheral vessel; and 3, central vessel. When a quadrant contained more than one vessel, the scores of the most severe vessels in that quadrant were calculated. Therefore, the maximum score was 6 for one quadrant region and 24 for the entire cornea^43^.
8. *Lid margin irregularity* Using a slit-lamp microscope, irregularities at the upper and lower eyelid margins were examined and photographed. The irregularities were graded as follows: 0, no lid margin irregularity; 1, less than three irregularities with shallow notches; 2, three or more irregularities with deep notches^42^. The scores of the upper and lower eyelids were summed to give a total score.
9. *Plugging of gland orifices* Using a slit-lamp microscope, the distribution of gland orifices at the gland orifice margin was examined and photographed. The examination was graded as follows: 0, no plugging of Mos; 1, fewer than three pluggings of Mos; 2, three or more pluggings of Mos, distributed over less than half the full length of the eyelid; 3, three or more pluggings of Mos, distributed over one-half (or more) of the eyelid’s full length^42^. The scores of the upper and lower eyelids were summed to give a total score.

### Marx’s line

The lid margin was stained using a lissamine green test strip to evaluate the Marx’s line. According to the score grading system of Yamaguchi et al.^44^, the Marx’s line scores were obtained for the outer, middle, and inner thirds of each palpebral margin. The scoring was conducted on a scale of 0 to 3, with 9 points in total. The score interpretations are as follows: 0, completely on the conjunctival side of Mos; 1, part of Marx’s line in contact with Mos; 2, Marx’s line cuts across all Mos; 3, Marx’s line moves to the side of the eyelid margin of Mos. The scores of the upper and lower eyelids were summed to give a total score for the affected eyes.

### Demodex examination

Six eyelashes (three each from the top and bottom eyelids) were extracted from each patient’s lid margins and then examined under an optical microscope to detect the presence of Demodex mites^45^. Three eyelashes with dandruff were selected from each of the nasal, median, and temporal sides. The extracted eyelashes were laid on a slide and covered with a coverslip, and a small amount of oil was dropped onto the slide. The slides were then examined under the microscope. The microscopic examinations and mite counts were performed by technicians who were blinded to the patient’s information. Patients were subsequently classified according to the presence or absence of Demodex mites on their eyelashes.

### Data analysis

All data were analyzed using SPSS 17.0 (SPSS, Inc., Chicago, IL, USA). The Kolmogorov–Smirnov test was used to assess the normality of the data. For comparisons between the two groups, Kruskal–Wallis tests were employed for non-normally distributed data, while independent t-tests were used for normally distributed data. Results are expressed as mean ± standard deviation. The differences between groups were also evaluated using Mann–Whitney nonparametric U tests, and the results are reported as medians (25% quantile, 75% quantile). Differences in population information distribution were assessed using the chi-square test. Spearman’s rank correlation was utilized to assess the correlation between the variables. A *P* < 0.05 was considered statistically significant.

## Data Availability

The data used and/or analyzed during this research are presented in this published article.
